# Combined Effect of Maternal Vitamin D Deficiency and Gestational Diabetes Mellitus on Trajectories of Ultrasound-Measured Fetal Growth: A Birth Cohort Study in Beijing, China

**DOI:** 10.1155/2020/4231892

**Published:** 2020-03-30

**Authors:** Zheng Liu, Hui Liu, Xiangrong Xu, Shusheng Luo, Jue Liu, Chuyao Jin, Na Han, Hai-Jun Wang

**Affiliations:** ^1^Department of Maternal and Child Health, School of Public Health, Peking University, Beijing 100191, China; ^2^Medical Informatics Center, Peking University, Beijing 100191, China; ^3^Department of Epidemiology and Biostatistics, School of Public Health, Peking University, Beijing 100191, China; ^4^Tongzhou Maternal and Child Health Hospital of Beijing, 101101, China

## Abstract

**Objective:**

Few studies have examined whether maternal 25(OH)D deficiency and gestational diabetes mellitus (GDM) jointly affect fetal growth. We aimed to examine the separate and combined effects of maternal 25(OH)D deficiency and GDM on trajectories of fetal growth.

**Methods:**

We established a birth cohort (2016-2017) with 10,913 singleton pregnancies in Tongzhou Maternal and Child Health Hospital of Beijing, China. Maternal 25(OH)D deficiency (serum 25(OH)D concentration < 20.0 ng/mL) was detected, and GDM was diagnosed at 24~28 gestational weeks. Fetal growth was assessed by longitudinal ultrasound measurements of estimated fetal weight (EFW) and abdominal circumference (AC) from 28 gestational weeks to delivery, both of which were standardized as gestational-age-adjusted *Z*-score. A *k*-means algorithm was used to cluster the longitudinal measurements (trajectories) of fetal growth. Logistic regression models were used for estimating exposure-outcome associations and additive interactions.

**Results:**

We identified two distinct trajectories of fetal growth, and the faster one resembling the 90^th^ centile curve in the reference population was classified as excessive fetal growth. Maternal 25(OH)D deficiency and GDM were independently associated with an increased risk of excessive fetal growth. The combination of maternal 25(OH)D deficiency and GDM was associated with an increased risk of excessive fetal growth assessed by EFW *Z*-score (odds ratio (OR): 1.36; 95% confidence interval (CI): 1.15~1.62) and AC *Z*-score (OR (95% CI): 1.32 (1.11~1.56)), but the relative excess risks attributable to interaction were nonsignificant (*P* > 0.05).

**Conclusion:**

Maternal 25(OH)D deficiency and GDM may jointly increase the risk of excessive fetal growth. Interventions for pregnancies with GDM may be more beneficial for those with 25(OH)D deficiency than those without regarding risk of excessive fetal growth, if confirmed in a large sample.

## 1. Introduction

Excessive fetal growth elevates the risk of acute and long-term complications for mothers and their offspring [1, 2]. Although gestational diabetes mellitus (GDM) is an established risk factor [3], mothers who seemingly achieve adequate glycemic control continue to experience a greater risk of fetal overgrowth [4, 5]. Thus, GDM may not be the only stimulus that drives fetal overgrowth and other potential contributors remain to be explored.

Vitamin D deficiency might be one of such potential contributors due to the important functions of vitamin D in fetal growth, including lipolysis, adipogenesis, and cell proliferation [6, 7]. Maternal vitamin D deficiency, assessed by serum 25-hydroxyvitamin D (25(OH)D) [8], was shown to have a positive association with excessive fetal growth in some studies, but not others [9–18]. Moreover, most previous studies assessed fetal growth by using birth weight rather than ultrasound measurements. However, ultrasound-measured fetal growth in utero appears to be associated with children's long-term outcomes independent of birth weight [19, 20]. Birth weight also cannot reflect an excess deposition of fat in the abdomen of fetuses, which is relatively common and clinically important in diabetic pregnancies [21].

It is important to examine the combined effect of maternal 25(OH)D deficiency and GDM on excessive fetal growth for three reasons. First, maternal 25(OH)D deficiency and GDM might share similar pathways, i.e., modulating adipocyte and glucose metabolism, by which the risk of excessive fetal growth might be elevated [22, 23]. This underlying etiology makes it valuable to examine whether the combined effect of maternal 25(OH)D deficiency and GDM is larger than the sum of their separate effects on excessive fetal growth. Second, in addition to an increasing prevalence of GDM, it was estimated that 18~84% of pregnancies were in deficit of vitamin D [24]. If maternal 25(OH)D deficiency and GDM interact and augment the effect of one another, it would be useful in targeting a high-risk subpopulation. For example, interventions for pregnancies with GDM may be more beneficial for those with 25(OH)D deficiency than those without regarding risk of excessive fetal growth. Third, the findings might also illuminate clinical practice in current China and many other countries with similar contexts, where clinical workers have not been fully aware of the implications of screening for maternal 25(OH)D deficiency.

Therefore, in this study, we aimed to examine the separate and combined effects of maternal 25(OH)D deficiency and GDM on trajectories of ultrasound-measured fetal growth. We hypothesized that maternal 25(OH)D deficiency and GDM would jointly affect fetal growth, and the combined effect of maternal 25(OH)D deficiency and GDM on excessive fetal growth is larger than the sum of their separate effects.

## 2. Methods

### 2.1. Study Design and Participants

We established a birth cohort in Tongzhou Maternal and Child Health Hospital (39°N latitude) of Beijing, China, from January 2016 to December 2017. All pregnant women were recruited during their first prenatal visit to the hospital after confirmation of their early pregnancy. In the first prenatal visit, pregnant women were interviewed face to face by trained nurses to collect their sociodemographic characteristics, gynecological and obstetrical history, last menstrual period (LMP), and anthropometric measurements. Gestational age was estimated using ultrasound measurement of crown-rump length when its difference with the gestational age based on the self-reported LMP was 7 days or more. At 24~28 weeks of gestation, maternal 25(OH)D concentrations were detected and GDM was diagnosed. During the follow-up prenatal visits, we kept tracking ultrasound measurements of fetal growth which were in line with the recommendation of the Chinese guideline of prenatal care [25]. At birth, the date of birth, neonatal gender, and birth weight were recorded. For this study, we included women delivering live singletons without congenital malformations, and those with diabetes or hypertension before pregnancy were excluded. We finally included 10,913 mother-offspring dyads. The study was approved by the Ethic Committee of Peking University Health Science Centre (IRB00001052-18004).

### 2.2. Assessment of Maternal 25(OH)D Deficiency and GDM

Fasting, 1-hour, and 2-hour plasma was obtained from all participants during the 2-hour, 75 g oral glucose tolerance test between 24 and 28 gestational weeks. GDM was diagnosed if glucose concentrations at fasting ≥ 5.1 mmol/L, 1 hour ≥ 10.0 mmol/L, or 2 hour ≥ 8.5 mmol/L [26]. The fasting sera were also measured for 25(OH)D (25(OH)D_2_ plus 25(OH)D_3_) by the high-performance liquid chromatography mass spectrometry method. Serum 25(OH)D concentration less than 20.0 ng/mL was defined as maternal 25(OH)D deficiency [8].

### 2.3. Assessment of Fetal Growth Trajectories

Estimated fetal weight (EFW) was predicted as a function of ultrasound-measured abdominal circumference (AC) and head circumference (HC) as ln (EFW) = 5.084820–54.06633 × (AC/100)3–95.80076 × (AC/100)3 × ln (AC/100) + 3.136370 × (HC/100), where EFW is in g and AC and HC are in cm [27]. Then, both EFW and AC were converted to gestational-age-adjusted *Z*-score to assess fetal growth [27, 28].

We used clustering techniques, based on a *k*-means algorithm for longitudinal data (KML) [29], to identify homogeneous clusters of individual trajectories of fetal growth. As we had no a priori knowledge of the optimal number of distinct trajectory groups, we searched for a minimum of 2 groups to a possible maximum of 6 groups. For each possible number of groups, we reran the KML algorithm 1000 times to search for the most optimal cluster result. We used the Calinski-Harabasz index to evaluate clustering quality [29]. An increase in this index indicates greater separation between clusters and greater similarity within clusters, thereby indicating better clustering quality.

### 2.4. Assessment of Covariates

We reviewed relevant literature and used a directed acyclic graph to create a least biased estimate of the exposure-outcome association. The covariates included maternal age at delivery (<*35 years*, ≥*35 years*), education level (*high school or below*, *college*, and *university or above*), employment (*yes*, *no*), parity (*primipara*, *multipara*), macrosomia delivery history (*yes*, *no*), prepregnancy weight status (*overweight*/*obese*, *not overweight*/*obese*), maternal triglyceride level measured in the first trimester (*above or below the median value in the study population*), sampling season (*spring*, *summer*, *autumn*, and *winter*), and neonatal sex (*male*, *female*). Information of covariates was obtained at the first prenatal visit or routine follow-ups.

### 2.5. Statistical Analyses

We analyzed data in three steps. First, we described characteristics of the cohort using means and proportions by maternal 25(OH)D deficiency and GDM status. We also described characteristics of fetal growth trajectories and their correlations with risk of large-for-gestational-age birth (LGA).

Second, we examined whether maternal 25(OH)D deficiency and GDM were independently associated with trajectories of fetal growth by using logistic regression analyses. We presented both crude and adjusted odds ratios (ORs) and 95% confidence intervals (CIs).

Third, we examined whether the combined effect of maternal 25(OH)D deficiency and GDM was larger than the sum of their separate effects on trajectories of fetal growth. We created a 4-category variable: no maternal 25(OH)D deficiency and no GDM, maternal 25(OH)D deficiency without GDM, GDM without maternal 25(OH)D deficiency, and both 25(OH)D deficiency and GDM. OR*_ij_* was used to represent the OR in both maternal 25(OH)D deficiency and GDM status *i*, *j*. If maternal 25(OH)D deficiency is present, then *i* = 1; otherwise, *i* = 0. If GDM is present, then *i* = 1; otherwise, *i* = 0. We estimated 3 ORs (i.e., OR_11_, OR_10_, OR_01_) with the reference category (OR_00_) from the logistic regression analyses. We assessed the presence of interactions by calculating the relative excess risk due to interaction (RERI = OR_11_ − OR_10_ − OR_01_ + 1) and corresponding 95% CIs, which was proposed by Knol and VanderWeele [30]. A positive, negative, and no interactions were reflected by a RERI > 0, RERI < 0, and RERI = 0, respectively.

To test robustness of our findings, we further conducted sensitivity analyses in a subset of low-risk pregnancies (i.e., pregnancies without use of insulin, hypertensive disorders, low-birth-weight infant (birth weight < 2500 g), and preterm birth (*delivery before 37 gestational weeks*)).

Tests of statistical significance were two sided, with *P* value < 0.05 denoting significant results. Description and regression analyses were performed with SPSS version 24.0 (SPSS Inc., Chicago, IL), and KML analyses were performed with R version 3.3.2 (R Foundation).

## 3. Results

### 3.1. General Characteristics of Study Population

Table 1 shows the general characteristics of the 10,193 mother-offspring dyads by maternal 25(OH)D deficiency and GDM status. In total, the mean (standard deviation (SD)) concentration of serum 25(OH)D was 26.6 (10.8) ng/mL, with 30.7% of pregnant women detected as having 25(OH)D deficiency. The mean (SD) of fasting, 1-hour, and 2-hour blood glucose concentrations was 4.7 (0.4), 7.6 (1.6), and 6.7 (1.2) mmol/L, respectively, with 20.8% of pregnant women affected by GDM. The prevalence of maternal 25(OH)D deficiency in pregnancies with GDM was 27.9%.

### 3.2. Characteristics of Fetal Growth Trajectories

There was a median (range) of 2 (2~5) ultrasound measures from 28 gestational weeks to delivery for each subject. The KML algorithm identified two distinct clusters of homogeneous fetal growth trajectories as the best clustering quality. Concerning fetal growth assessed by EFW *Z*-score, the patterns distinguish one trajectory of faster fetal growth (accounting for 49.3%, mean increasing rate (SD) of EFW: 217.7 (39.2) g/week) from the other trajectory of slower fetal growth (50.7%, 186.0 (37.4) g/week); concerning fetal growth assessed by AC *Z*-score, the patterns distinguish one trajectory of faster fetal growth (47.2%, 9.9 (2.0) mm/week) from the other trajectory of slower fetal growth (52.8%, 9.4 (2.0) mm/week) (Supplemental [Supplementary-material supplementary-material-1]). The mean increasing rates of EFW and AC within the trajectories of slower and faster fetal growth resembled the 50^th^ and 90^th^ centile curves in the international standards for fetal growth, respectively [27, 28], and pregnancies in the trajectories of faster fetal growth were associated with an increased risk of LGA at birth (OR (95% (CI): 10.7 (9.2, 12.5) for fetal growth assessed by EFW *Z*-score; 7.9 (6.8, 9.0) for fetal growth assessed by AC *Z*-score) compared with those in the trajectories of slower fetal growth. Therefore, increases in EFW or AC were defined as excessive fetal growth if they followed the trajectories of faster fetal growth that were classified by the KML algorithm in this study.

### 3.3. Associations of Maternal 25(OH)D Deficiency and GDM with Fetal Growth Trajectories

Table 2 describes the associations of maternal 25(OH)D deficiency and GDM with odds of membership in trajectories of faster fetal growth (excessive fetal growth). Maternal 25(OH)D deficiency was independently associated with excessive fetal growth assessed by EFW *Z*-score. GDM was independently associated with excessive fetal growth assessed by EFW *Z*-score or AC *Z*-score. These results were unaltered when restricting the sample in a subset of low-risk pregnancies (Supplemental [Supplementary-material supplementary-material-1]).

### 3.4. Separate and Combined Effects of Maternal 25(OH)D Deficiency and GDM on Fetal Growth Trajectories

Table 3 describes the separate and combined effects of maternal 25(OH)D deficiency and GDM on the odds of membership in trajectories of faster fetal growth (excessive fetal growth). Pregnancies with 25(OH)D deficiency and GDM in combination significantly elevated the risk of excessive fetal growth assessed by EFW *Z*-score (OR (95% CI): 1.36 (1.15, 1.62) or AC *Z*-score (1.32 (1.11, 1.56)) compared with pregnancies without 25(OH)D deficiency and GDM. The observed associations remained robust when restricting analyses in a low-risk subpopulation (Supplemental [Supplementary-material supplementary-material-1]). The combined effects of maternal 25(OH)D deficiency and GDM were slightly larger than the sum of their separate effects on excessive fetal growth assessed by EFW *Z*-score or AC *Z*-score, but the additive interactions were not significant (RERI: 0.11 (-0.16, 0.38) for EFW *Z*-score, 0.08 (-0.19, 0.34) for AC *Z*-score).

## 4. Discussion

Our findings suggest that maternal 25(OH)D deficiency and GDM were independently associated with an increased risk of excessive fetal growth. Moreover, the combination of 25(OH)D deficiency and GDM was associated with an increased risk of excessive fetal growth. The additive interactions between 25(OH)D deficiency and GDM on the risk of excessive fetal growth were in the expected direction (i.e., positive interaction), but not statistically significant. To our knowledge, our study is the first to examine the combined effect of maternal 25(OH)D deficiency and GDM in relation to trajectories of ultrasound-measured fetal growth.

Previous data suggest that 25(OH)D modulates adipocyte Ca^2+^ signaling and therefore might exert a coordinated control over lipogenesis and lipolysis [22]. Additionally, specific receptors of 25(OH)D have been found in pancreatic *β* cells in the human and rat pancreases [31], which suggested a possible role of 25(OH)D in maintaining normal glucose homeostasis [22, 23]. These mechanisms suggested that maternal 25(OH)D deficiency might share similar pathways (i.e., modulating adipocyte and glucose metabolism) with GDM in elevating the risk of excessive fetal growth and could interpret the observed combined effect of maternal 25(OH)D deficiency and GDM on excessive fetal growth in our study population.

Our findings should be interpreted cautiously due to the potential weaknesses as in most observational studies. First, we may not directly control residual confounders such as diet and physical activity. We did, however, take into account maternal lipid levels which reflect maternal lifestyle during pregnancy, thus making our results less likely biased. Second, despite the best available approach we have taken to fully use the cohort data, we cannot test the level of vitamin D in the first trimester due to the practical issue, providing the possibility for a future study. Third, we found that although the observed combined effect of maternal 25(OH)D deficiency and GDM on excessive fetal growth was slightly larger than would have been expected on the additive scale, the RERI was not statistically significant. A pragmatic explanation for the nonsignificant interaction is the relatively small sample size for the subgroup analysis.

Despite these, this study specifically addressed some important limitations of previous studies. First, we assessed fetal growth by using ultrasound measures of EFW and AC, while previous studies typically utilized birth weight, which cannot adequately capture dynamic growth in utero. Moreover, birth weight cannot reflect the asymmetric increases in AC, which is clinically important in diabetic pregnancies. Second, we used serum 25(OH)D concentration (rather than dietary report) as a reliable indicator of vitamin D status, which quantifies both the outdoor exposure and dietary intake of vitamin D. Third, considering the increasing prevalence of maternal 25(OH)D deficiency and GDM, it would be of public health importance to examine their combined effect in relation to excessive fetal growth, which is useful in targeting a high-risk subpopulation.

## 5. Conclusion

Maternal 25(OH)D deficiency and GDM may jointly increase the risk of excessive fetal growth. Interventions for pregnancies with GDM may be more beneficial for those with 25(OH)D deficiency than those without regarding risk of excessive fetal growth, if confirmed in a large sample.

## Figures and Tables

**Table 1:** Characteristics of study population by maternal 25(OH)D deficiency and GDM status.

Characteristics	All (*N* = 10,913)	No VDD and no GDM (*n* = 5928)	VDD without GDM (*n* = 2714)	GDM without VDD (*n* = 1637)	Both VDD and GDM (*n* = 634)	*P* value^∗^
Maternal age at delivery, *n* (%)						
<35 years	9857 (90.3)	5385 (90.8)	2528 (93.1)	1386 (84.7)	558 (88.0)	<0.001
≥35 years	1056 (9.7)	543 (9.2)	186 (6.9)	251 (15.3)	76 (12.0)	
Maternal education levels, *n* (%) (missing = 178)						
High school or below	3041 (28.3)	1591 (27.2)	769 (29.2)	444 (27.4)	237 (38.2)	<0.001
College	3370 (31.4)	1810 (30.9)	895 (33.9)	480 (29.7)	185 (29.8)	
University or above	4324 (39.6)	2457 (41.9)	974 (36.9)	694 (42.9)	199 (32.0)	
Employment, *n* (%)						
Yes	9405 (86.2)	5086 (85.8)	2373 (87.4)	1395 (85.2)	551 (86.9)	0.12
No	1508 (13.8)	842 (14.2)	341 (12.6)	242 (14.8)	83 (13.1)	
Parity, *n* (%)						
Primipara	6688 (61.3)	3628 (61.2)	1718 (63.3)	961 (58.7)	381 (60.1)	0.02
Multipara	4225 (38.7)	2300 (38.8)	996 (36.7)	676 (41.3)	253 (39.9)	
Macrosomia delivery history, *n* (%)						
Yes	150 (1.4)	74 (1.2)	29 (1.1)	38 (2.3)	9 (1.4)	0.004
No	10763 (98.6)	5854 (98.8)	2685 (98.9)	1599 (97.7)	625 (98.6)	
Prepregnancy BMI status, *n* (%) (missing = 262)						
Overweight/obese	2412 (22.1)	1137 (19.6)	542 (20.7)	494 (30.6)	239 (38.5)	<0.001
Not overweight/obese	8239 (75.5)	4662 (80.4)	2073 (79.3)	1123 (69.4)	381 (61.5)	
Maternal TG level, median (IQR) (mmol/L)	1.0 (0.8~1.3)	0.9 (0.7~1.2)	1.0 (0.7~1.2)	1.1 (0.8~1.4)	1.1 (0.8~1.5)	<0.001
Sampling season, *n* (%)						
Spring	2972 (27.2)	1469 (24.8)	885 (32.6)	407 (24.9)	211 (33.3)	<0.001
Summer	2826 (25.9)	1936 (32.7)	321 (11.8)	492 (30.1)	77 (12.1)	
Autumn	2436 (22.3)	1389 (23.4)	484 (17.8)	446 (27.2)	117 (18.5)	
Winter	2679 (24.5)	1134 (19.1)	1024 (37.7)	292 (17.8)	229 (36.1)	
Hypertensive disorders of pregnancy, *n* (%)						
Yes	457 (4.2)	219 (3.7)	101 (3.7)	93 (5.7)	44 (6.9)	<0.001
No	10456 (95.8)	5709 (96.3)	2613 (96.3)	1544 (94.3)	590 (93.1)	
Newborn sex, *n* (%)						
Male	5574 (51.1)	2996 (50.5)	1413 (52.1)	842 (51.4)	323 (50.9)	0.61
Female	5339 (48.9)	2932 (49.5)	1301 (47.9)	795 (48.6)	311 (49.1)	

^∗^ *χ* ^2^ test or independent samples of the Kruskal-Wallis test. Abbreviations: GDM: gestational diabetes mellitus; IQR: interquartile range; TG: triglyceride; VDD: maternal 25(OH)D deficiency.

**Table 2:** Associations of maternal 25(OH)D deficiency and GDM with fetal growth trajectories.

Predictors	*N*	Fetal growth assessed by EFW *Z*-score			Fetal growth assessed by AC *Z*-score		
		*n* (%) with excessive fetal growth	Crude OR	Adjusted OR	*n* (%) with excessive fetal growth	Crude OR	Adjusted OR
VDD							
No	7565	3662 (48.4)	Reference	Reference	3538 (46.8)	Reference	Reference
Yes	3348	1717 (51.3)	1.12 (1.04, 1.22)	1.11 (1.02, 1.21)	1618 (48.3)	1.07 (0.98, 1.16)	1.06 (0.97, 1.15)
GDM							
No	8642	4157 (48.1)	Reference	Reference	3958 (45.8)	Reference	Reference
Yes	2271	1222 (53.8)	1.26 (1.15, 1.38)	1.17 (1.06, 1.29)	1198 (52.8)	1.32 (1.20, 1.45)	1.20 (1.09, 1.32)

Abbreviations: AC: abdominal circumference; EFW: estimated fetal growth; CI: confidence interval; GDM: gestational diabetes mellitus; OR: odds ratio; VDD: maternal 25(OH)D deficiency.

**Table 3:** Separate and combined effects of maternal 25(OH)D deficiency and GDM on fetal growth trajectories.

Predictors	*N*	Fetal growth assessed by EFW *Z*-score			Fetal growth assessed by AC *Z*-score		
		*n* (%) with excessive fetal growth	Crude OR	Adjusted OR	*n* (%) with excessive fetal growth	Crude OR	Adjusted OR
No VDD, no GDM	5928	2798 (47.2)	Reference	Reference	2686 (45.3)	Reference	Reference
VDD without GDM	2714	1359 (50.1)	1.12 (1.02, 1.23)	1.11 (1.00, 1.21)	1272 (46.9)	1.07 (0.97, 1.17)	1.05 (0.95, 1.16)
GDM without VDD	1637	864 (52.8)	1.25 (1.12, 1.40)	1.15 (1.03, 1.29)	852 (52.0)	1.31 (1.17, 1.46)	1.19 (1.06, 1.33)
Both VDD and GDM	634	358 (56.5)	1.45 (1.23, 1.71)	1.36 (1.15, 1.62)	346 (54.6)	1.45 (1.23, 1.71)	1.32 (1.11, 1.56)

Abbreviations: AC: abdominal circumference; EFW: estimated fetal growth; CI: confidence interval; GDM: gestational diabetes mellitus; OR: odds ratio; VDD: maternal 25(OH)D deficiency.

## Data Availability

The datasets generated during and/or analyzed during the current study are not publicly available but are available from the corresponding author on reasonable request.

## Supplementary Materials

Supplementary Materials Supplemental Table 1: associations of maternal 25(OH)D deficiency and GDM with fetal growth trajectories (sensitivity analyses in low-risk cases). Supplemental Table 2: separateand combined effects of maternal 25(OH)D deficiency and GDM on fetal growth trajectories (sensitivity analyses inlow-risk cases). Supplemental Figure 1: fetal growth trajectories in estimated fetal weight and abdominal circumference.
