# Supplementary material for: Combined Effect of Maternal Vitamin D Deficiency and Gestational Diabetes Mellitus on Trajectories of Ultrasound-Measured Fetal Growth: A Birth Cohort Study in Beijing, China
Source: J Diabetes Res. 2020 Mar 30;2020:4231892. doi: 10.1155/2020/4231892 (PMC7149432; doi:10.1155/2020/4231892)
Supplement: Supplementary Materials — Supplemental Table 1: associations of maternal 25(OH)D deficiency and GDM with fetal growth trajectories (sensitivity analyses in low-risk cases). Supplemental Table 2: separateand combined effects of maternal 25(OH)D deficiency and GDM on fetal growth trajectories (sensitivity analyses inlow-risk cases). Supplemental Figure 1: fetal growth trajectories in estimated fetal weight and abdominal circumference. [file 4231892.f1.docx]

**Online Supporting Material**

- **List of the supplementary tables**

**1. Supplemental Table 1** Associations of maternal 25(OH)D deficiency and GDM with fetal growth trajectories (sensitivity analyses in low-risk cases)

**2. Supplemental Table 2** Separate and combined effects of maternal 25(OH)D deficiency and GDM on fetal growth trajectories (sensitivity analyses in low-risk cases)

**3.** **Supplemental Figure 1** Fetal growth trajectories in estimated fetal weight and abdominal circumference

**Supplemental Table 1** Associations of maternal 25(OH)D deficiency and GDM with fetal growth trajectories (sensitivity analyses in low-risk cases)

| **Predictors** | **N** | **Fetal growth assessed by EFW Z-score** | | | **Fetal growth assessed by AC Z-score** | | |
| --- | --- | --- | --- | --- | --- | --- | --- |
|  |  | **n (%) with excessive fetal growth** | **Crude OR** | **Adjusted OR** | **n (%) with excessive fetal growth** | **Crude OR** | **Adjusted OR** |
| **VDD** |  |  |  |  |  |  |  |
| No | 7103 | 3471 (48.9) | Reference | Reference | 3351 (47.2) | Reference | Reference |
| Yes | 3165 | 1632 (51.6) | 1.11 (1.02, 1.21) | 1.09 (1.00, 1.20) | 1537 (48.6) | 1.06 (0.97, 1.15) | 1.04 (0.95, 1.14) |
| **GDM** |  |  |  |  |  |  |  |
| No | 8219 | 3989 (48.5) | Reference | Reference | 3802 (46.3) | Reference | Reference |
| Yes | 2049 | 1114 (54.4) | 1.26 (1.15, 1.39) | 1.19 (1.08, 1.32) | 1086 (53.0) | 1.31 (1.19, 1.44) | 1.21 (1.10, 1.34) |

Abbreviations: AC = abdominal circumference; EFW = estimated fetal growth; CI = confidence interval; GDM = gestational diabetes mellitus; OR = odds ratio; VDD = maternal 25(OH)D deficiency.

**Supplemental Table 2** Separate and combined effects of maternal 25(OH)D deficiency and GDM on fetal growth trajectories (sensitivity analyses in low-risk cases)

| **Predictors** | **N** | **Fetal growth assessed by EFW Z-score** | | | **Fetal growth assessed by AC Z-score** | | |
| --- | --- | --- | --- | --- | --- | --- | --- |
|  |  | **n (%) with excessive fetal growth** | **Crude OR** | **Adjusted OR** | **n (%) with excessive fetal growth** | **Crude OR** | **Adjusted OR** |
| No VDD, no GDM | 5629 | 2681 (47.6) | Reference | Reference | 2578 (45.8) | Reference | Reference |
| VDD without GDM | 2590 | 1308 (50.5) | 1.12 (1.02, 1.23) | 1.09 (0.99, 1.21) | 1224 (47.3) | 1.06 (0.97, 1.16) | 1.04 (0.94, 1.15) |
| GDM without VDD | 1474 | 790 (53.6) | 1.27 (1.13, 1.43) | 1.18 (1.05, 1.33) | 773 (52.4) | 1.31 (1.16, 1.46) | 1.20 (1.07, 1.35) |
| Both VDD and GDM | 575 | 324 (56.3) | 1.42 (1.19, 1.69) | 1.35 (1.12, 1.61) | 313 (54.4) | 1.41 (1.19, 1.68) | 1.30 (1.08, 1.55) |

Abbreviations: AC = abdominal circumference; EFW = estimated fetal growth; CI = confidence interval; GDM = gestational diabetes mellitus; OR = odds ratio; VDD = maternal 25(OH)D deficiency.


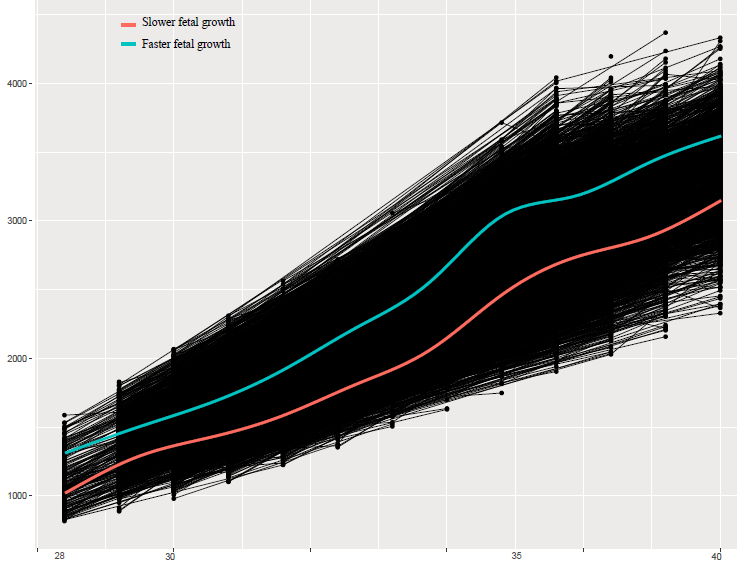

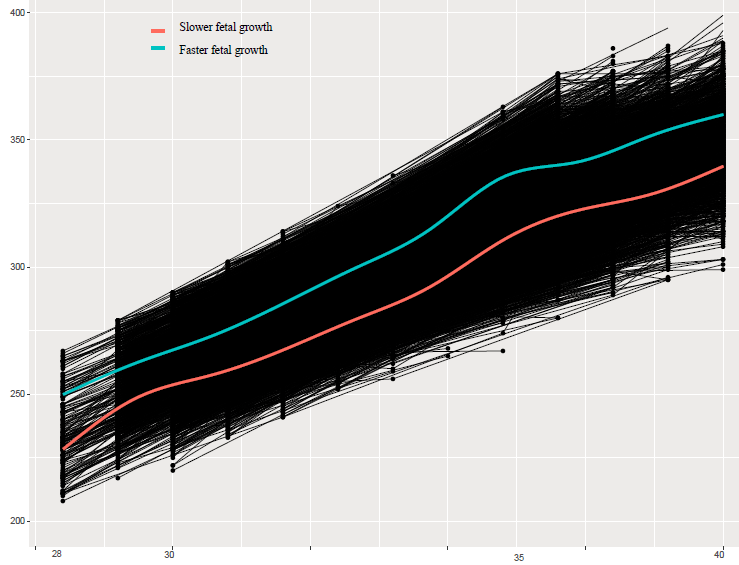


**(B)**

**(A)**

Abdominal circumference (mm)

(g)

Estimated fetal growth (g)

Weeks of gestation Weeks of gestation

**Supplemental Figure 1 Fetal growth trajectories in estimated fetal weight (A) and abdominal circumference (B)**

(The lines refers to distinct homogeneous fetal growth trajectories in terms of estimated fetal weight (Figure (A)) and abdominal circumference (Figure (B)) based on individual ultrasound measurements (blue line: faster fetal growth; red line: slower fetal growth). Over follow-up, longitudinal ultrasound measurements collected for each fetus can be seen as trajectories. We used clustering techniques, based on k-means algorithm, to build summaries (lines) that reveal patterns for homogeneous groups of fetal growth trajectories.)
